# Antiseptic Injections and Drainage Tubes in Empyema

**Published:** 1872-12-15

**Authors:** Edmund Andrews

**Affiliations:** Prof. of Surgery in Chicago Medical College


Antiseptic Injections and Drainage Tubes in Empyema.—By Edmund Andrews, M. D. Prof. of Surgery in Chicago Medical College.

The great success of carbolic acid injections in arresting the purulent secretion, and consequent exhaustion and hectic of large abscesses determined me to try it in Empyema. The following case shows the success:

Michael Hennessy was stabbed in the back part of the left chest, last June. The wound was closed, but effusion occurred, which became purulent, and discharged through a fistulous orifice, remaining after the tapping of the cavity. A few months later he was found confined to the bed, emaciated, and slowly going down with the copious suppuration.

My first effort was to inject carbolated water into the fistula; but I found the opening small and crooked, and the interior so full of pus that not enough of the antiseptic could be gotten in to accomplish anything. I then anæsthetized the patient and made a good sized opening into the chest between the ribs where the fistula lay. About a quart of pus gushed out, showing that the contraction of the orifice had kept in the discharge, and prevented the tendency of the cavity to gradually collapse and become obliterated. To prevent a recurrence of the trouble, I inserted a piece of small rubber tube, and tied it in. Through this tube large injections of carbolated water, ten grains to the ounce, were freely thrown once a day. Between the dressings the wound and the orifice of the tube were kept guarded by a compress of cotton dipped in carbolated oil (40 grs. to the ounce.) By this treatment the secretion of pus has been almost arrested; the patient is growing fat and vigorous, and is already able to take long walks. The cavity is becoming smaller, and bids fair to be safely obliterated.

Experiments made by Dr. Andrews, in Mercy Hospital, show that although Cundurango has no control over cancerous tumors, it powerfully promotes the growth of granulations in chronic indolent ulcers. He promises shortly to give us his facts in full.

The U. S. Government has adopted for Marine Hospital use the ecraseur forceps, invented by Dr. E. Andrews, of this city, as a substitute for the old-fashioned chain ecraseur.

Why does'nt some one give us a condensed statement of the medical literature of Japan?

---

Subcutaneous Injection of Mercury in Syphilis.—The injection of bichloride of mercury in the treatment of syphilis finds a warm advocate in Dr. Staub, of Strasbourg (*Traitement de la Syphilis*), who specially recommends the following formula: Bichloide of mercury, 1.25 *grammes;* chloride of ammonium, 1.25 *grammes;* chloride of sodium, 4.15 *grammes;* the white of one egg; distilled water, 250 *grammes;* One *centigramme* of this is injected daily beneath the skin, in two portions, one-half in the morning, the other in the evening. According to this somewhat enthusiastic writer, the bichloride, so administered, is an invaluable therapeutic agent, infallible in the treatment of secondary symptoms, prompt, complete, and devoid of inconvenience.— *Gaz. Hebdom.*

